# Detection of Genomic Regions Associated with Resistance to Stem Rust in Russian Spring Wheat Varieties and Breeding Germplasm

**DOI:** 10.3390/ijms21134706

**Published:** 2020-07-01

**Authors:** Irina N. Leonova, Ekaterina S. Skolotneva, Elena A. Orlova, Olga A. Orlovskaya, Elena A. Salina

**Affiliations:** 1 The Federal Research Center Institute of Cytology and Genetics SB RAS, 630090 Novosibirsk, Russia; skolotnevaES@bionet.nsc.ru (E.S.S.); orlovaea@bionet.nsc.ru (E.A.O.); salina@bionet.nsc.ru (E.A.S.); 2 Institute of Genetics and Cytology of the National Academy of Sciences of Belarus, 220072 Minsk, Belarus; flaxol@tut.by

**Keywords:** wheat, *Triticum aestivum*, Puccinia graminis, introgression lines, GWAS, *Sr* genes

## Abstract

Stem rust caused by *Puccinia graminis* f. sp. *tritici* Eriks. is a dangerous disease of common wheat worldwide. Development and cultivation of the varieties with genetic resistance is one of the most effective and environmentally important ways for protection of wheat against fungal pathogens. Field phytopathological screening and genome-wide association study (GWAS) were used for assessment of the genetic diversity of a collection of spring wheat genotypes on stem rust resistance loci. The collection consisting of Russian varieties of spring wheat and introgression lines with alien genetic materials was evaluated over three seasons (2016, 2017 and 2018) for resistance to the native population of stem rust specific to the West Siberian region of Russia. The results indicate that most varieties displayed from moderate to high levels of susceptibility to *P. graminis*; 16% of genotypes had resistance or immune response. In total, 13,006 single-nucleotide polymorphism (SNP) markers obtained from the Infinium 15K array were used to perform genome-wide association analysis. GWAS detected 35 significant marker-trait associations (MTAs) with SNPs located on chromosomes 1A, 2A, 2B, 3B, 5A, 5B, 6A, 7A and 7B. The most significant associations were found on chromosomes 7A and 6A where known resistance genes *Sr25* and *Sr6Ai = 2* originated from *Thinopyrum* ssp. are located. Common wheat lines containing introgressed fragments from *Triticum timopheevii* and *Triticum kiharae* were found to carry *Sr36* gene on 2B chromosome. It has been suggested that the quantitative trait loci (QTL) mapped to the chromosome 5BL may be new loci inherited from the *T. timopheevii*. It can be inferred that a number of Russian wheat varieties may contain the *Sr17* gene, which does not currently provide effective protection against pathogen. This is the first report describing the results of analysis of the genetic factors conferring resistance of Russian spring wheat varieties to stem rust.

## 1. Introduction

Stem rust of wheat caused by the biotrophic fungus *Puccinia graminis* f. sp. *tritici* Erikss. & Henning (*Pgt*) is one of the highly devastating diseases of cereals, which results in the destruction of susceptible plants under conditions favorable for the development of the infection. The distribution area, race composition and virulence of the pathogen vary significantly depending on the region of wheat cultivation and climate features. A historically significant spread and harmfulness of the pathogen have been observed in North and South America, Australia, Africa and China [1,2]. However, in the second half of the 20th century, the deleterious effect of stem rust in these countries decreased. This was due to the creation of new wheat varieties with genetic resistance to stem rust [3]. Measures developed for destruction of barberry as an intermediate host in the pathogen development cycle also had a significant impact [4].

Over the past two decades, the epidemiological situation regarding this disease has deteriorated significantly. Epidemics and sporadic outbreaks of stem rust are currently recorded in Western Europe [5]. The emergence of a new aggressive *Pgt* race Ug99, rapidly spreading across the African continent, resulted in crop losses of up to 80–100% [6]. The Ug99 race and its biotypes (Ug99 + *Sr36* and Ug99 + *Sr24*) are now registered in 13 countries: Egypt, Ethiopia, Eritrea, Iran, Kenya, Mozambique, Rwanda, Sudan, South Africa, Tanzania, Uganda, Yemen and Zimbabwe [7,8,9].

In the Russian Federation, stem rust was extremely rare in the regions of the North Caucasus and the Volga region, while the disease was focal and had no significant economic consequence. Currently, the pathogen distribution area has expanded, and crop damage is observed in almost all regions of Russia [10,11,12]. This is largely due to climate changes, pathogen migration dynamics, the emergence of new virulence races and the loss of resistance of wheat varieties to *Pgt*. Monitoring of the race composition of the stem rust in the Central region of Russia showed a significant diversity of *P. graminis* with the dominance of highly virulent pathotypes [13]. Strong development of stem rust, close to epiphytotic, has been observed since 2016 in the Republic of Tatarstan, while all wheat cultivars recommended for cultivation in the region turned out to be susceptible to the pathogen [14].

The Ural and West Siberian regions are the largest producers of spring bread wheat, which accounts for more than 40% of the acreage. However, despite the tendency to increase the productivity, there is a variation in the yield over the years, which largely depends on the resistance of commercial wheat cultivars to fungal diseases. In the Ural and Siberian regions, stem rust was not registered previously, but, since 2008, it is observed annually. In the past few years, pathogen development in certain areas has reached the level of epiphytoty with crop damage up to 80% and yield reduction up to 30–40% [12,15,16]. A pronounced increase in the pathogen virulence and aggressiveness has been observed in recent years in Northern Kazakhstan, adjacent to the West Siberian region [17].

In connection with the change in the phytosanitary status for stem rust in the Siberian region, constant monitoring of the race composition of the *Pgt* population and assessment of the susceptibility of wheat varieties recommended for cultivation in the region are required. The study and monitoring of the stem rust race composition in the Siberian region was launched in 2016. A comparison of the race biotypes of pathogens from different areas of Western Siberia suggests the existence of the Omsk and Altai subpopulations of *P. graminis*. At the same time, wheat crop areas in the Novosibirsk region are located in the migration zone between subpopulations and their mixing [18].

The development and use of resistant wheat varieties should be preceded by an analysis of the genetic basis of resistance. Currently, more than 80 stem rust (*Sr*) resistance genes have been identified in the genome of common wheat and wheat relatives [19]. The effectiveness of these genes in relation to local *Pgt* populations and Ug99 biotypes was evaluated [20,21,22]. Molecular markers for the identification of *Sr* genes and for marker-assisted selection (MAS) were developed (https://maswheat.ucdavis.edu/). The emergence of new virulent pathotypes, as well as the loss of efficiency of known *Sr* genes, generate a need for finding new sources of resistance for stem rust control.

The dynamic development of the West Siberian population of the fungus and the features of its race composition emphasize the importance for screening of wheat varieties and the search for donors of resistance loci. The aim of this work was to study a collection consisting of cultivars and breeding lines of spring bread wheat and promising common wheat introgression lines (ILs) with alien genetic material. The collection was not previously characterized by susceptibility to the stem rust pathogen and there is no information on the genetic factors that could determine disease resistance.

## 2. Results

### 2.1. Phenotyping 

Weather conditions during the years of testing were favorable for the development of stem rust infection. In 2016 and 2017, the first symptoms of the infection were recorded at the beginning of the second decade of July; in 2018, the infection appeared only at the end of the third decade of July, when early ripening varieties entered the stage of milky ripeness. The severity rating (SR) of the universally susceptible cultivar Chernyava-13 was 60MS–70S (Table S1) on the Cobb’s scale, suggesting that the infection level was sufficient for a clear scoring of plant response. Analysis of variance (ANOVA) performed over three years of testing showed significant differences among genotypes and years of evaluation (Table 1).

**Figure 1:**
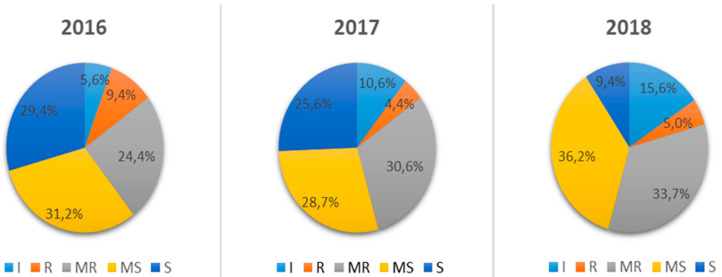
Diagram of distribution of spring wheat varieties by susceptibility to stem rust during 2016–2018.

The analysis of the distribution of the infection patterns over the three seasons showed that on average 54% of the genotypes exhibited a highly and moderately susceptible types of the reaction (SR > 10MS–5S), while 16% were resistant genotypes (SR < 10R) (Figure 1 and Table S1). According to monitoring data, the pathogen background in different areas of Western Siberia was higher during 2016–2017 and the infection appeared much earlier [12,18]. It resulted in a three-fold increase in the number of susceptible genotypes compared to 2018. It is necessary to emphasize the peculiarities of the development of stem rust in the region, which consist in a low degree of severity rating (SR > 10–30%) and a highly sensitive type of the reaction. It is due to an early decrease in the average daily temperature and the transition of the fungus to a stage of teliospores, which are unable to further infect wheat.

**Figure 2:**
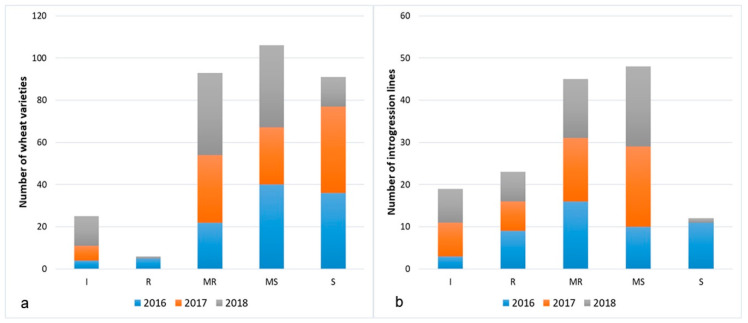
Frequencies of wheat varieties (**a**) and introgression lines (**b**) in different infection types to stem rust pathogen.

The comparison of the severity ratings of commercial varieties and introgression lines showed that the number of genotypes characterized by a resistant type of the reaction was significantly higher among ILs (Figure 2a,b). Thus, the immune and resistant (IT = 0/R) types of the reaction in all three years displayed 49% of ILs and only 15% of wheat cultivars and breeding lines.

### 2.2. Genetic Population Structure

**Figure 3:**
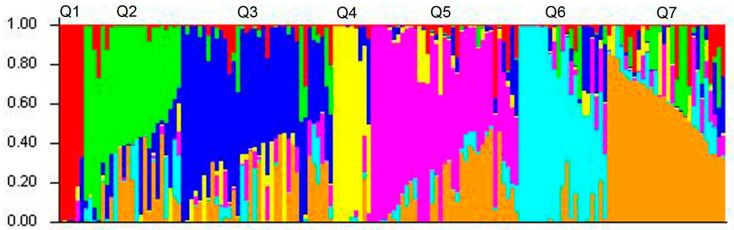
The population structure of spring wheat varieties calculated based on genotyping using 4800 SNP markers. The list of the genotypes belonging to the individual subpopulation is presented in Table S2.

Four thousand eight hundred SNP markers covering all chromosomes of common wheat were used to determine the genetic population structure. Based on the results obtained using the STRUCRURE and ΔK statistics, there were seven postulated subclusters, including 6, 24, 35, 9, 35, 21 and 28 genotypes, respectively (Figure 3 and Table S2).

Clusters 1 and 4 included *T. aestivum*/*T. timopheevii* introgression lines obtained by hybridization with wheat varieties Tselinnaya-20 and Irtyshanka-10, respectively. Clusters 2 and 7 consisted of Russian wheat varieties developed in various Russian breeding companies. Clusters 3 and 6 consisted mainly of Russian varieties; besides, ILs Saratovskaya-29/*T. timopheevii* and Novosibirskaya-67/*T. timopheevii* combined with three and six subpopulations, respectively. A set of ILs obtained with the participation of *T. durum*, *T. dicoccum*, *T. dicoccoides* and *T. kiharae*, as well as the original parental forms, grouped in Cluster 5. The dendrogram of genetic similarity, which was constructed using the complete set of SNP markers by the method of nearest neighbors, largely supported the results from the Bayesian-based clustering (Figure S1).

### 2.3. Marker-Trait Association Study

**Figure 4:**
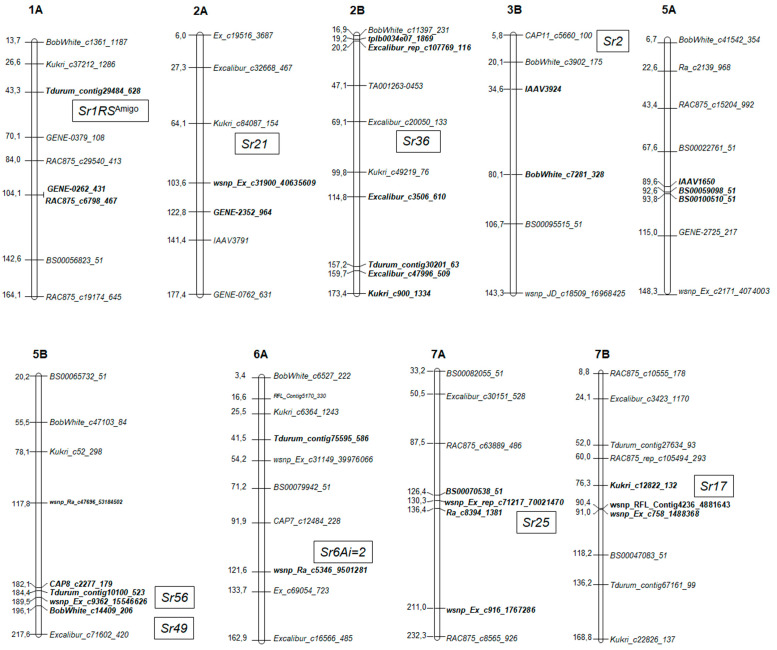
Schematic illustration of the localization of loci determining resistance to stem rust in wheat genotypes. Markers identified in this paper are indicated in bold. The order of the markers corresponds to the consensus chromosome maps for the SNP [23]. Known *Sr* resistance genes were added at the right side of the chromosomes according to published data [19].

For genotyping of plant material, 13,006 SNP markers were used; after filtering, the number of markers for association mapping was 10,924 (Table S3). The number of markers mapped to different chromosomes of the A, B or D genomes varied significantly; the smallest one was observed for the fourth homoeological group. To perform the genome-wide association study, the mixed linear model (MLM) was used, which took into account the population structure (Q) and kinship (K). The quantile–quantile plot, illustrating the correspondence between the observed and expected *p*-values for MLM, is shown in Figure S2. Association mapping revealed 84 marker-trait associations (MTAs) for resistance to stem rust. The most informative SNPs (*p* < 0.005) were combined in nine QTLs on chromosomes 1A, 2A, 2B, 3B, 5A, 5B, 6A, 7A and 7B according to consensus genetic maps of wheat [23,24] (Table 2 and Figure 4).

Significant associations on chromosomes 6A and 6D were detected in five varieties: Tulaikovskaya-zolotistaya, Tulaikovskaya-10, Lutescens-101, Kinelskaya-60 and Volgouralskaya. It should be noted that the favorable allele of the marker wsnp_Ra_c5346_9501281 (*p* = 7.02 × 10^−4^) was amplified only in the genomes of three cultivars (Tulaikovskaya-zolotistaya, Tulaikovskaya-10 and Lutescens-101), while the favorable alleles Tdurum_contig75595_586 (*p* = 1.01 × 10^−3^) and Excalibur_rep_c99143_422 (*p* = 2.36 × 10^−3^) mapped at 6AS and 6DS were found in all five varieties (Table 2).

At the long arm of chromosome 7A, QTL was identified, which is associated with the resistance of varieties Tulaikovskaya-belozernaya, Tulaikovskaya-1, Volgouralskaya, Kinelskaya-60, Erithrospermum-72, Albidum-73 and Strada-Sibiri. Three SNPs (Ra_c8394_1381, BS00070538_51 and wsnp_Ex_rep_c71217_70021470) are located in the range from 126.40 to 136.43 cM; the wsnp_Ex_c916_1767286 (*p* = 9.38 × 10^−4^) occupies the position 211.00 cM at the distal part of the long arm (Figure 4). Minor QTL was detected at the long arm of chromosome 7B in the range of 76–91 cM on consensus map. Favorable alleles of three SNP markers (wsnp_RFL_Contig4236_4881643 and wsnp_Ex_c758_1488368, Kukri_c12822_132) with a probability of *p* < 0.00269–0.00358 were found in wheat varieties Altaiskii-prostor, Obskaya-14, Polyushko, Baganskaya-93, Rybinskaya-127, Salimovka, Nostalgiya, Ustya, Zlatozara and Obskaya-2.

Markers GENE_0262_431, RAC875_c6798_467 and Tdurum_contig29484_628 that showed association with resistance to stem rust were located at short arm of 1A chromosome. Favorable alleles amplified in the genome of eight wheat varieties (Ustya, Omskaya-20, Priirtyshskaya-86, Pitic-62, Rybinskaya-127, Katyusha, Tarskaya-6 and Tertsiya) (Table 2). Reliable MTAs were found for nine SNP markers mapped to chromosome 2B. Three SNPs (tplb0034e071869, Excalibur_rep_c107769_116 and Kukri_c46621_143) are mapped in the regions of 19.15–20.16 cM and 88.86 of the short arm; four markers are localized on the long arm, three of these SNPs (Excalibur_c47996_509, Tdurum_contig30201_63 and Kukri_c900_1334) was mapped in the range from 157.21 to 173.35 cM. For markers wsnp_Ex_c20169_29215401 and wsnp_Exc20169_29215401, the exact positions on the chromosome are unknown. Favorable alleles were recorded in all *T. aestivum*/*T. timopheevii* introgression lines, in line 25-2, containing the genetic material of *T. kiharae*, and in parental wheats *T. timopheevii* and *T. kiharae* (Table 2).

On chromosome 5BL, five reliable MTAs were detected in the region from 182.14 to 196.08 cM. All SNP markers amplify favorable alleles in line 25-2, in most *T. aestivum*/*T. timopheevii* ILs, with the exception of the lines derived from the variety Irtyshanka-10. The genetic locus on chromosome 5AL contributes to the resistance of ILs 221-1 (*T. durum*/Belorusskaya 80), 16-5 (*T. dicoccoides*/Festivalnaya), varieties Pitic-62 and Rybinskaya-127. This is evidenced by reliable MTAs for SNP markers BS00059098_51, BS00100510_51, IAAV1650 mapped in the region 89.56–93.75 cM, and marker Ra_c14657_919 with an unknown position at the chromosome.

Associations with resistance response have been established on 2AL chromosome for two SNPs: the favorable allele wsnp_Ex_c31900_40635609 was detected in ILs with *T. timopheevii* genetic material, while resistance allele of GENE-2352_964 was amplified in lines 206-2, 213-1, 226-7, 25-2, 34-1 and 16-5, obtained by hybridization with *T. kiharae* and *T. dicoccoides*. Minor marker-trait associations were identified for the BobWhite_c7281_328 and IAAV3924 markers mapped to the short arm of 3B chromosome in the regions of 80.12 and 34.61 cM, respectively (Figure 4). Favorable alleles were amplified in the genomes of 11 wheat varieties and 4 ILs (Table 2).

## 3. Discussion

Little is known about the genetic resistance to stem rust in Russian bread wheat varieties thus far. Meanwhile, there is an urgent need to search for new sources and donors of stem rust resistance genes. Studies for identification of *Sr* genes were performed primarily on collections of synthetic wheats containing the *Ae. tauschii* genome and common wheat lines with introgressions from *Aegilops* ssp. [12,15,22]. The screening carried out in these studies using molecular markers designed for known *Sr* genes showed that the genotypes contain mainly the *Sr25*, *Sr31*, *Sr24* and *Sr17* genes and their combinations. In this study, the genetic diversity of *Sr* loci was estimated in the germplasm of spring wheat varieties suitable for release in Western Siberia. Screening material was extended with introgression lines containing the genetic material from the species of *Triticeae* tribe.

To search for stem rust resistance loci, genome-wide association study was applied, which allowed identifying QTLs on chromosomes 1A, 2A, 2B, 3B, 6A, 5A, 5B, 7A and 7B. At present, GWAS is an effective method for assessing the genetic diversity of crops on disease resistance genes, for studying the genetic architecture of agronomically important traits and for determining the chromosomal localization of valuable genes and quantitative trait loci [25,26,27,28]. Employing this approach, it is possible to analyze large size populations using the results obtained in different environments [29,30]. GWAS allows us to postulate the presence of both known resistance genes and previously unidentified loci. For example, new loci of resistance to stem rust, including Ug99, were found with SNP genotyping of large collections of spring wheat varieties and breeding lines of various origins [31,32,33].

In our work, the postulation of the genetic loci was made by comparing the GWAS results with published data on the chromosomal location of known *Sr* genes. The positions of loci at the chromosome were established in accordance with the consensus maps of hexaploid wheat constructed using a 90K SNP array. Significant marker-trait associations on chromosomes 6A and 6D were found in Tulaikovskaya-zolotistaya, Tulaikovskaya-10, Lutescens-101, Volgouralskaya and Kinelskaya-60 varieties, which displayed a high level of resistance in all years of trait evaluation (Table S1). According to the literature data, varieties Tulaikovskaya-zolotistaya, Tulaikovskaya-10 and Lutescens-101 contain the substitution of chromosome 6D on chromosome 6Ai = 2 from *Th. intermedium* harboring genes for resistance to fungal diseases [34,35]. The resistance of these varieties to *Pgt* seems to be determined by the *Sr6Ai = 2* gene.

It has been suggested that the resistance of six varieties (Tulaikovskaya-belozernaya, Kinelskaya-60, Volgouralskaya, Erithrospermum-72, Albidum-73 and Strada-Siberi) is determined by the *Sr25* gene inherited from chromosome 7Ae#1L of another wildgrass species *Th. ponticum* [36]. This is evidenced by associations with SNP markers located in the range from 126 to 211 cM at the long arm of chromosome 7A. Additionally, *Lr19* gene tightly linked to *Sr25* was previously obtained in the genome of Kinelskaya-60 and Volgouralskaya varieties [37].

SNP markers specific to chromosome 2B showed associations with the resistance of all *T. aestivum*/*T. timopheevii* introgression lines and line 25-2 (*T. aestivum*/*T. kiharae*). The distribution of reliable MTAs along the length of the entire chromosome may indicate complete 2B/2G substitution in these genotypes. The results suggest that ILs contain the *Sr36* gene introduced from *T. timopheevii* [38]. Genotypes with *Sr36* gene are known to express high level of resistance to stem rust, including the Ug99 race [39,40]. The results of the field screening obtained in this work may also indicate the effectiveness of the *Sr36* gene against the local *Pgt* population.

Marker trait associations at the long arm of chromosome 5B were detected in lines 25-2 and 34-1 (*T. kiharae*/Saratovskaya-29) and in *T. aestivum*/*T. timopheevii* lines, with the exception of ILs originating from the Irtyshanka-10 variety (Table 2). At the telomeric region of 5ВL, two adult plant resistance genes were mapped—*Sr49* (range 188.57–217.64 cM) and *Sr56* (159.66–188.67 cM)—inherited from the winter wheat variety Arina and landrace variety Mahmoudi [41,42]. *T. aestivum*/*T. timopheevii* lines possessed a 5BS.5BL.5GL telomeric translocation with the *LrTt2* gene [43]. It is possible that the translocation fragment harbors a new genetic factor from 5G chromosome of *T. timopeevii* and *T. kiharae*, which determines resistance to *Pgt*.

Significant MTAs were detected for three SNP located on chromosome 1A (Table 2). A set of QTLs at the long arm of 1A chromosome were identified using mapping populations and GWAS [31,44]. However, the position of these loci does not coincide with the locus identified in this work. *Sr1RS*^Amigo^ gene was introgressed into chromosome 1A of bread wheat from rye *Secale cereale* [19]. The analysis of the pedigree of the varieties Omskaya-20, Priirtyshskaya-86 and Ustya indicates the participation of the cultivars Kavkaz and Bezostaya-1 in their creation. This may be indirect evidence of the presence of the *Sr1RS* gene or its allele on chromosome 1A.

The long arm of chromosome 5A harbors QTL, located in the range of 89.56–93.75 cM. No stem rust resistance genes with constant symbols are currently mapped on chromosome 5A. Bajgain et al. [31] and Letta et al. [45] reported the presence of genetic factors for resistance to *Pgt* in this chromosome. However, the localization of these loci does not coincide with the QTL position established in our work.

Genetic factor on chromosome 7B was identified in the range 76.3–91.02 cM (Figure 4). According to data of various authors, the *Sr17* gene was mapped in the diapason from 64.7 to 127 cM using bi-parental mapping populations and GWAS [44,46,47]. This allowed us to suggest that the QTL at chromosome 7B may be the *Sr17* gene.

At chromosome 2A, MTAs were detected for two SNPs located at a distance of 20 cm from each other. It is important to note that these loci are associated with the resistance of different genotypes: marker wsnp_Ex_c31900_40635609 was detected in ILs obtained from *T. timopheevii* and *T. kiharae*, the favorable allele of GENE-2352_964 was found in the genome of ILs containing alien chromatin from *T. dicoccum* and *T. dicoccoides*. *Sr21* gene originated from *T. monococcum* was mapped before at the long arm of chromosome 2A [48,49]. It is possible that alleles of this gene were introduced into the ILs from tetraploid relatives.

Two marker trait-associations were detected at the short arm of chromosome 3B. Chromosome 3BS bears the slow rusting gene *Sr2*; additionally, a number of QTLs was found at 3BS, which are probably to be *Sr2* [31,44,45]. In this study, QTL at 3BS was detected in wheat varieties, which displayed significant variability in stem rust severity from 5R to 80S during 2016–2017. Since the physiological marker of *Sr2*, known as “pseudo-black chaff” [50], was not phenotyped among these varieties, we hypothesized the novel QTL that could provide the slow rusting effect here. Taking into account that not only host resistance but environmental effect could result in slow-rusting, it is necessary to measure the area under the disease progress (AUDPC) before assuming the certain type of resistance for varieties.

## 4. Materials and Methods

### 4.1. Plant Material and Phenotyping

Plant material consisted of 158 wheat varieties, among them 105 spring wheat cultivars and advanced breeding lines adapted for cultivation to Siberian region, 53 introgression lines (ILs) and their parental forms (Table S1). Seeds of spring wheat cultivars and breeding lines, obtained from the National Genebank of Russian Federation (VIR, Federal Research Center N.I. Vavilov All-Russian Institute of Plant Genetic Resources, St. Petersburg, Russia; http://db.vir.nw.ru/virdb/maindb), were maintained and multiplied in The Federal Research Center Institute of Cytology and Genetics SB RAS (IC&G SB RAS, Novosibirsk, Russia). Introgression lines were developed in the Institute of Cytology and Genetics SB RAS (Novosibirsk, Russia) and in the Institute of Genetics and Cytology (Minsk, Republic of Belarus) on the base of hybridization of spring bread wheat varieties with wild relatives (*T. durum*, *T. dicoccum*, *T. dicoccoides* and *T. timopheevii*) and synthetic hexaploid wheat *T. kiharae* [51,52]. The experimental plants were grown on the field of the Federal Research Center Institute of Cytology and Genetics SB RAS (Novosibirskaya oblast, 54.9191° N, 82.9903° E). Samples were sown in a randomized block design in two replicates on plots 1 m wide, 60 grains per row. Stem rust severity was evaluated against a natural infectious background during the summer season 2016–2018 (from June to August), from the onset of the first symptoms of the disease to the full development of the disease. Disease severity (SR) was estimated on a 0–100% modified Cobb scale [53]. Infection response (IT) was recorded as recommended by Roelfs et al. [1].

### 4.2. Genotyping and Statistical Analysis

Genomic DNA was isolated from 5–7-day-old seedlings as described in Kiseleva et al. [54]. Genotyping was carried out with the help of the Illumina Infinium 15K array of TraitGenetics GmbH (Gatersleben, Germany, www.traitgenetics.de), which included 13,006 SNP markers mapped in the wheat genome [23,24].

Analysis of variance (ANOVA) of the data on stem rust resistance in different environments was performed using the program STATISTICA v. 10 (www.statsoft.ru). The population structure (Q-matrix) was estimated using a Bayesian algorithm implemented in the program STRUCTURE 2.3.4 [55]. Q-matrix was calculated based on the results of genotyping with 4800 SNP markers. The number of suspected subclusters ranged from 1 to 10. The simulation was performed using the admixture model; the number of runs was five with a burn-in length of 20,000 and Markov chain iterations of 50000. The most likely number of clusters was calculated from Delta K (ΔK) statistics [56] using the web-based program Structure Harvester [57]. Kinship (K) matrix was calculated using the program TASSEL V. 5.2.50 [58]. A complete set of SNP markers was used to calculate the K-matrix, with the exception of markers that showed missing data for all analyzed samples.

Marker-trait associations (MTAs) were determined on the basis of mixed linear model (MLM) with kinship matrix (K) and population structure (Q) as covariate using the program TASSEL v. 5.2.50. SNP markers with MAF (minor allele frequency) less than 5% and missing data > 10% were not included in the analysis. To identify reliable MTAs, Benjamini–Hochberg method [59] was used for controlling false discovery rate (FDR) of *p* < 0.05. FDR was calculated by means of False Discovery Rate Online Calculator (https://tools.carbocation.com/FDR). The proposed genetic location of QTLs associated with stem rust resistance was determined using consensus maps of hexaploid wheat chromosomes presented by Wang et al. [23]. Chromosome maps were constructed with the MapChart version 2.3 software [60].

## 5. Conclusions

The results received in this study show the effectiveness of using GWAS to assess the genetic basis of resistance of wheat genotypes to stem rust pathogen. Obtained results allow us to estimate genetic protection level of cultivated spring wheat varieties and breeding germplasm. Nevertheless, it should be noted that, for a clear postulation of known and new resistance loci, additional analysis is needed using molecular markers developed for *Sr* genes. Moreover, to establish the chromosomal localization of target genes in the genome of donors containing alien translocations, cytological analysis is required. The obtained data can be considered as the initial stage for the selection of introgression lines with alien genetic material as sources of *Sr* loci.

## Figures and Tables

**Table 1:** Analysis of variance of stem rust resistance in spring wheat genotypes.

	DF	SS	MS	F value	*p*
Genotype	159	491.447	3.110	2.846	0.000000
Environment	2	23.258	11.629	10.639	0.000034
Error	318	345.409	1.093		

**Table 2:** Marker-trait associations for stem rust resistance in spring bread wheat genotypes.

SNP Marker	Chr *	Allele	Distance *	*p*	R^2^	Genotype
GENE-0262_431	1A	**A**/G	104.145	3.67 × 10^−4^	0.192	Ustya, Omskaya-20, Priirtyshskaya-86, Pitic-62, Rybinskaya-127, Katyusha, Tarskaya-6, Tertsiya
RAC875_c6798_467		**G**/A	104.145	1.15 × 10^−3^	0.164	
Tdurum_contig29484_628		**C**/T	43.27	2.37 × 10^−3^	0.129	
wsnp_Ex_c31900_40635609	2A	**C**/A	103.62	1.69 × 10^−3^	0.152	ILs (821, 832, 837, 842, 157, 38, 67, 94, 140, 199, 676, 25-2, 34-1, 15-7), *T. timopheevii*, *T. kiharae*, *T. dicoccoides*
GENE-2352_964		**G**/A	122.82	1.88 × 10^−3^	0.150	ILs (206-2, 213-1, 226-7, 25-2, 34-1, 16-5), Pitic-62, *T. kiharae*
wsnp_Ex_c20169_29215401	2B	**A**/C	nd	9.24 × 10^−4^	0.158	ILs (*T. aestivum*/*T. timopheevii*, 25-2), *T. timopheevii*, *T. kiharae*
tplb0034e07_1869		**C**/T	19.15	2.81 × 10^−3^	0.201	
Excalibur_rep_c107769_116		**A**/G	20.16	1.63 × 10^−3^	0.168	
Kukri_c46621_143		**T**/C	88.86	2.62 × 10^−4^	0.245	
Excalibur_c3506_610		**T**/C	114.81	1.83 × 10^−3^	0.217	
wsnp_Ex_c20169_29215401		**C**/A	nd	1.08 × 10^−3^	0.238	
Excalibur_c47996_509		**A**/G	159.65	1.13 × 10^−3^	0.201	
Tdurum_contig30201_63		**G**/T	157.21	3.43 × 10^−3^	0.159	
Kukri_c900_1334		**T**/C	173.35	2.63 × 10^−3^	0.169	
BobWhite_c7281_328	3B	**C**/T	80.12	1.87 × 10^−3^	0.143	Tulaikovskaya-1, Lutescens-85, Lutescens-148, Altaiskaya-530, Erythrospermum-72, Novosibirskaya-22, Lutescens-25, Obskaya-14, Krasa-2, Rybinskaya-127, Pitic-62, ILs (213-1, 221-1, 34-1, 16-5)
IAAV3924		**C**/T	34.61	3.27 × 10^−3^	0.187	
Ra_c14657_919	5A	**A**/G	nd	1.12 × 10^−3^	0.201	Rybinskaya-127, Pitic-62, ILs (221-1, 16-5), *T. dicoccoides*
BS00059098_51		**C**/A	92.60	2.63 × 10^−3^	0.193	
BS00100510_51		**G**/T	93.75	3.23 × 10^−3^	0.196	
IAAV1650		**A**/G	89.56	4.61 × 10^−3^	0.149	
CAP8_c2277_179	5B	**T**/C	182.14	8.77 × 10^−6^	0.298	ILs (Saratovskaya-29/*T. timopheevi*, Skala/*T. timopheevii*, Novosibirskaya-67/*T. timopheevii*, 34-1, 25-2), *T. timopheevii*, *T. kiharae*
wsnp_Ex_c9362_15546626		**G**/A	189.51	2.24 × 10^−5^	0.245	
Tdurum_contig10100_523		**G**/A	184.39	4.43 × 10^−5^	0.231	
wsnp_Ra_c47696_53184502		**A**/G	117.85	6.49 × 10^−4^	0.200	
BobWhite_c14409_206		**T**/C	196.08	7.11 × 10^−4^	0.209	
wsnp_Ra_c5346_9501281	6A	**T**/C	121.61	7.02 × 10^−4^	0.203	Tulaikovskaya-zolotistaya, Tulaikovskaya-10, Lutescens-101
Tdurum_contig75595_586		**G**/A	41.46	1.01 × 10^−3^	0.174	Tulaikovskaya-zolotistaya, Tulaikovskaya-10, Kinelskaya-60, Volgouralskaya, Lutescens-101
Excalibur_rep_c99143_422	6D	**C**/A	22.96	2.36 × 10^−3^	0.156	
Ra_c8394_1381	7A	**A**/G	136.43	6.86 × 10^−5^	0.129	Tulaikovskaya-belozernaya, Kinelskaya-60, Volgouralskaya, Erythrospermum-72, Albidum-73, Srada-Sibiri
BS00070538_51		**T**/C	126.4	2.78 × 10^−4^	0.192	
wsnp_Ex_rep_c71217_70021470		**C**/A	130.27	9.79 × 10^−4^	0.124	
wsnp_Ex_c916_1767286		**C**/T	211.00	9.38 × 10^−4^	0.168	
wsnp_RFL_Contig4236_4881643	7B	**G**/A	90.36	2.69 × 10^−3^	0.191	Altaiskii-prostor, Obskaya-14, Polushko, Baganskaya-93, Rybinskaya-127, Salimovka, Nostalgiya, Ustya, Zlatozara, Obskaya-2
wsnp_Ex_c758_1488368		**C**/T	91.02	2.85 × 10^−3^	0.189	
Kukri_c12822_132		**G**/A	76.3	4.72 × 10^−3^	0.114	

* Chromosomal localization and marker position on the chromosome are indicated according to the consensus maps of *T. aestivum* L. [23,24]; favorable alleles are highlighted in bold; R^2^ indicates phenotypic variation explained by the significant locus.

## Supplementary Materials

Supplementary materials can be found at https://www.mdpi.com/1422-0067/21/13/4706/s1.

## Abbreviations

ANOVA	Analysis of variance
GWAS	Genome-wide association study
ILs	Introgression lines
MAS	Marker assisted selection
MLM	Mixed linear model
MTA	Marker trait association
*Pgt*	*Puccinia graminis* f. sp. *tritici*
QTL	Quantitative trait locus
SR	severity rating
SNP	Single-nucleotide polymorphism
*Sr* gene	Stem rust resistance gene
