# Supplementary material for: Detection of Genomic Regions Associated with Resistance to Stem Rust in Russian Spring Wheat Varieties and Breeding Germplasm
Source: Int J Mol Sci. 2020 Jul 1;21(13):4706. doi: 10.3390/ijms21134706 (PMC7369787; doi:10.3390/ijms21134706)
Supplement: Supplementary file 1 [file ijms-21-04706-s001.zip › Table S3.pdf]

Table S3. Number of SNP markers with localizations in the A, B and D genomes used for association mapping.

Genome	Chromosome							
	1	2	3	4	5	6	7	Total
A	605	555	545	446	644	638	704	4137
B	799	1054	786	333	911	847	565	5295
D	299	419	138	46	176	182	232	1492
