# Supplementary figures and images for: Detection of Genomic Regions Associated with Resistance to Stem Rust in Russian Spring Wheat Varieties and Breeding Germplasm
Source: Int J Mol Sci. 2020 Jul 1;21(13):4706. doi: 10.3390/ijms21134706 (PMC7369787; doi:10.3390/ijms21134706)

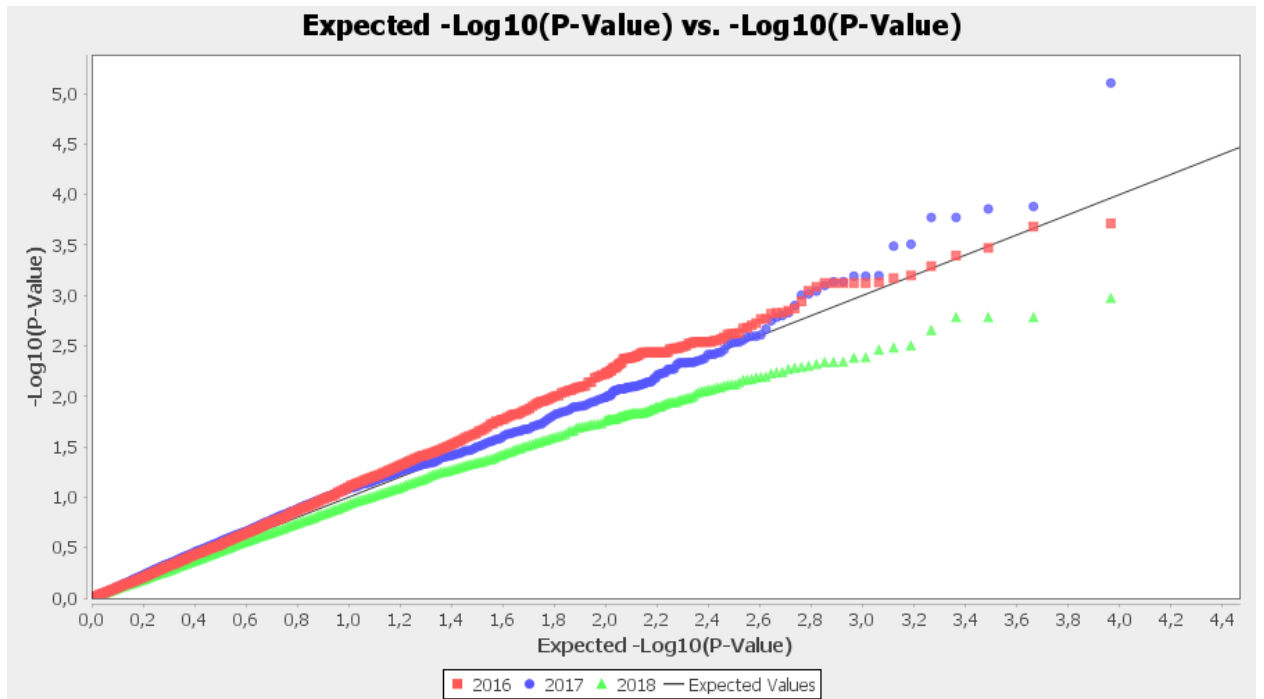

Figure S2. The quantile-quantile plot for MLM model.

Supplement: Supplementary file 1 [file ijms-21-04706-s001.zip › Figure S2.pdf]
